# HBV Core Protein Enhances Cytokine Production

**DOI:** 10.3390/diseases3030213

**Published:** 2015-09-17

**Authors:** Tatsuo Kanda, Shuang Wu, Reina Sasaki, Masato Nakamura, Yuki Haga, Xia Jiang, Shingo Nakamoto, Osamu Yokosuka

**Affiliations:** Department of Gastroenterology and Nephrology, Graduate School of Medicine, Chiba University, 1-8-1 Inohana, Chuo-ku, Chiba 260-8670, Japan; E-Mails: gosyou100@yahoo.co.jp (S.W.); reina_sasaki_0925@yahoo.co.jp (R.S.); masato.nm@hotmail.co.jp (M.N.); hagayuki@gmail.com (Y.H.); jxia925@yahoo.co.jp (X.J.); nakamotoer@yahoo.co.jp (S.N.); yokosukao@faculty.chiba-u.jp (O.Y.)

**Keywords:** HBV, core, cytokine, interferon

## Abstract

Hepatitis B virus (HBV) infection, a cause of hepatocellular carcinoma (HCC), remains a serious global health concern. HCC development and human hepatocarcinogenesis are associated with hepatic inflammation caused by host interferons and cytokines. This article focused on the association between the HBV core protein, which is one of the HBV-encoding proteins, and cytokine production. The HBV core protein induced the production of interferons and cytokines in human hepatoma cells and in a mouse model. These factors may be responsible for persistent HBV infection and hepatocarcinogenesis. Inhibitors of programmed death (PD)-1 and HBV core and therapeutic vaccines including HBV core might be useful for the treatment of patients with chronic HBV infection. Inhibitors of HBV core, which is important for hepatic inflammation, could be helpful in preventing the progression of liver diseases in HBV-infected patients.

## 1. Introduction

Hepatitis B virus (HBV) infection can lead to acute and chronic hepatitis, cirrhosis and hepatocellular carcinoma (HCC) and is a global health concern [1]. Although the HBV vaccine and drugs, such as peginterferon and nucleos(t)ide analogues (NUCs), are effective against HBV infection, further investigation is needed to eradicate HBV [2,3].

HBV is a hepatotropic virus, belonging to the *Hepadnaviridae* family, with a circular, partially-double-stranded DNA of approximately 3.2 kb in length [4]. On the shorter plus stranded DNA, at least four genes (*surface* (*S*), *core* (*C*), *X* and *polymerase* (*P*)) are encoded, and these genes partially overlap [4]. The *Pre-S* gene, which consists of the *pre-S1* and *pre-S2* genes, is located upstream of the S gene. The S gene encodes an envelope protein, HBV surface antigen (HBsAg), which comprises 226 amino acids. The function of the HBx protein in the HBV life cycle remains unclear [5]. It is reported that HBx is associated with hepatocarcinogenesis [6]. HBV polymerase (P) protein is important for HBV replication. HBV polymerase encodes the RNA- and DNA-dependent DNA polymerase [5]. The *Pre-C* region is located upstream of the *C* gene. HBV *pre-C* and *C* regions encode both HBV core genes (183 codons) and HBeAg (149 codons) [4,7].

HBV core protein self-assembles to form the viral capsid [8]. The functions of HBV core protein during the life cycle of HBV or in HBV infection of human liver are not well understood [8]. Liver cirrhosis is the strongest risk factor for the development of HBV-related HCC [9]. The production of inflammatory cytokines, such as TNF-α and TGF-β, is linked to hepatic fibrosis and hepatocarcinogenesis [9]. In this article, we focus on the association between HBV core protein and cytokine production.

## 2. HBV Core Protein and Cytokine Production

HBV replicates in human hepatocytes. We examined whether HBV core protein enhances 84 interferon- and cytokine-related gene expression levels in the human hepatoma cell line HepG2 with stable expression of HBV core (HepG2-HBV core) compared to a HepG2 control by a real-time RT-PCR-based array (toll-like receptor signaling pathway PCR array, Qiagen, Hilden, Germany) [7,9]. After transfection of the plasmids and three weeks of G418 selection, to avoid monoclonal selection, all cells were collected, and these cells, HepG2-HBV core and HepG2 control, were established for further analysis. Out of 84 genes, only eight (9.5%) genes [nuclear factor of kappa light polypeptide gene enhancer in B-cells 2 (p49/p100) (NFKB2), tumor necrosis factor receptor superfamily, member 1A (TNFRSF1A), ubiquitin-conjugating enzyme E2 variant 1 (UBE2V1), myeloid differentiation primary response 88 (MYD88), prostaglandin-endoperoxide synthase 2 (PTGS2), toll-like receptor adaptor molecule 1 (TICAM1), ECSIT signaling integrator (ECSIT) and interleukin-1 receptor-associated kinase 1 (IRAK1)] were downregulated by at least 1.2-fold in the HepG2-HBV core compared to the HepG2 control, and four of them (PTGS2, TICAM1, ECSIT and IRAK1) were downregulated by greater than 10-fold or more. Out of 84 interferon- and cytokine-related genes, 76 (90.5%) were upregulated by 1.2-fold or greater in the HepG2-HBV core cells compared to the HepG2 control. Fifty-eight genes were upregulated 50-fold or more in HepG2-HBV core cells (Table 1). Our results showed that HBV core protein seems involved in the induction of several interferon- and cytokine-related genes.

**Table 1:** Interferon- and cytokine-related genes upregulated by at least 50-fold in HepG2-HBV core cells analyzed using real-time RT-PCR.

Symbol	Name
***CD180***	CD180 molecule
***IL8***	Chemokine (C-X-C motif) ligand 8
***IL6***	Interleukin 6
***CLEC4E***	C-type lectin domain family 4, member E
***TLR10***	Toll-like receptor 10
***TLR8***	Toll-like receptor 8
***IFNG***	Interferon, gamma
***TLR2***	Toll-like receptor 2oll-like receptor 2
***IL12A***	Interleukin 12A
***MAPK8***	Mitogen-activated protein kinase 8
***LY96***	Lymphocyte antigen 96
***IFNB1***	Interferon, beta 1, fibroblast
***TNF***	Tumor necrosis factor
***CHUK***	Conserved helix-loop-helix ubiquitous kinase
***NFKB1***	Nuclear factor of kappa light polypeptide gene enhancer in B-cells 1
***TLR3***	Toll-like receptor 3
***IFNA1***	Interferon, alpha 1
***PRKRA***	Protein kinase, interferon-inducible double stranded RNA dependent activator
***NFKBIA***	Nuclear factor of kappa light polypeptide gene enhancer in B-cells inhibitor, alpha
***NR2C2***	Nuclear receptor subfamily 2, group C, member 2
***IL2***	Interleukin 2
***ELK1***	ELK1, member of ETS oncogene family
***TBK1***	TANK-binding kinase 1
***LY86***	Lymphocyte antigen 86
***HMGB1***	High mobility group box 1
***TLR7***	Toll-like receptor 7
***IRF3***	Interferon regulatory factor 3
***RIPK2***	Receptor-interacting serine-threonine kinase 2
***TOLLIP***	Toll interacting protein
***IKBKB***	Inhibitor of kappa light polypeptide gene enhancer in B-cells, kinase beta
***IRAK2***	Interleukin-1 receptor-associated kinase 2
***PELI1***	Pellino E3 ubiquitin protein ligase 1
***MAP3K7***	Mitogen-activated protein kinase kinase kinase 7
***TRAF6***	TNF receptor-associated factor 6, E3 ubiquitin protein ligase
***REL***	v-rel avian reticuloendotheliosis viral oncogene homolog
***CD86***	CD86 molecule
***FOS***	FBJ murine osteosarcoma viral oncogene homolog
***MAP2K4***	Mitogen-activated protein kinase kinase 4
***CASP8***	Caspase 8, apoptosis-related cysteine peptidase
***EIF2AK2***	Eukaryotic translation initiation factor 2-alpha kinase 2
***SARM1***	Sterile alpha and TIR motif containing 1
***CSF3***	Colony stimulating factor 3 (granulocyte)
***MAP4K4***	Mitogen-activated protein kinase kinase kinase kinase 4
***CXCL10***	Chemokine (C-X-C motif) ligand 10
***UBE2N***	Ubiquitin-conjugating enzyme
***MAP3K1***	Mitogen-activated protein kinase kinase kinase 1, E3 ubiquitin protein ligase
***NFRKB***	Nuclear factor related to kappaB binding protein
***HRAS***	Harvey rat sarcoma viral oncogene homolog
***IL1B***	Interleukin 1, beta
***JUN***	Jun proto-oncogene
***IL10***	Interleukin 10
***PPARA***	Peroxisome proliferator-activated receptor alpha
***CD80***	CD80 molecule
***RELA***	v-rel avian reticuloendotheliosis viral oncogene homolog A
***MAPK8IP3***	Mitogen-activated protein kinase 8 interacting protein 3
***HSPD1***	Heat shock 60 kDa protein 1 (chaperonin)
***TLR1***	Toll-like receptor 1
***TLR9***	Toll-like receptor 9

## 3. HBV Infection Induces Cytokine Production

**Figure 1:**
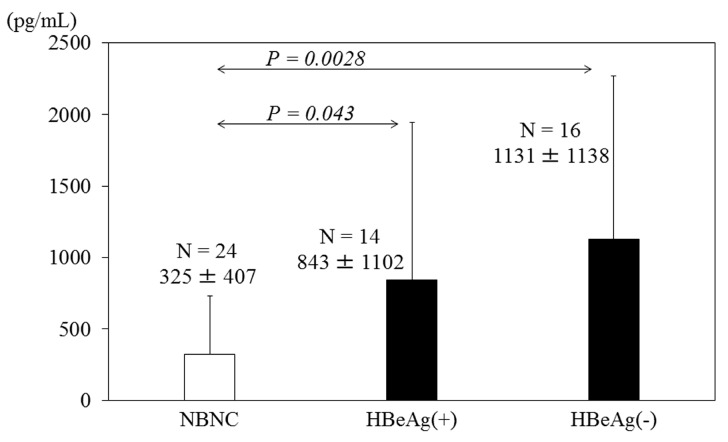
Serum IL-6 levels in patients with normal ALT levels. IL-6 levels were determined by enzyme-linked immunosorbent assay (ELISA; KOMABIOTECH, Seoul, Korea) following the manufacturer’s protocol. Non-B, non-C (NBNC), patients without HBV or HCV infection (males, *n* = 15; mean age, 57 ± 14 years); HBe antigen (HBeAg)(+), patients with HBeAg-positive asymptomatic carriers (males, *n* = 6; mean age, 34 ± 15 years); HBeAg(−), patients with HBeAg-negative asymptomatic carriers (males, *n* = 7; mean age, 51 ± 14 years).

TNF-α and the interferon system are important for HBV clearance [10,11,12,13,14,15]. Tzeng *et al.* [15] reported that HBV core is critical for inducing TNF-α to clear HBV and for TNF inhibition, which eliminates HBV core-induced viral clearance effects in mice. We also found that IL6 protein production was much higher in cell culture medium of HepG2-HBV core than in the HepG2 control [7,9]. Serum IL6 levels are higher in patients infected with HBV than those without HBV (Figure 1). Of interest, the IL6 level of HBeAg-positive patients is less than that of HBeAg-negative patients, although these patients had normal ALT levels. We also reported that the inflammatory cytokines, such as IL6, were downregulated in HBeAg-positive HepG2, which stably expresses HBeAg, compared to HBeAg-negative HepG2 cells [7,9].

Lin *et al.* [16] found that C57BL/6 mice injected with mutant HBV DNA did not express the HBV core gene and that approximately 90% of the established HBsAg persisted after six months. Conversely, C57BL/6 mice administered a single intravenous hydrodynamic injection of the HBV DNA without mutation exhibited HBV replication, which resulted in approximately 20%–30% of these mice carrying HBV for more than six months. Lin *et al.* [16] reported that the C-terminal domain of HBV core is necessary for HBV clearance. HBV core protein may influence HBV persistence by evoking innate immunity through the binding of the C-terminal domain of HBV core to membrane heparin sulfate on the surfaces of immune cells, which stimulates proinflammatory cytokine production [16].

## 4. Targeting Therapies and Vaccines against HBV Core

Thus, the HBV core protein seems to play an important role in persistent HBV infection. Therefore, targeting therapies against HBV core also look important as one of the new treatment options against HBV infection. Treatment with monoclonal antibodies against the immunologic receptor programmed death (PD)-1, a 55-kDa transmembrane protein, reversed the impairment of HBV core-specific interferon-γ T-cell response in C57BL/6 mice with HBV persistence [17]. Tzeng *et al.* [17] found that knock-out HBcAg, but not HBeAg, led to HBV persistent infection in mice and reported that the immune response triggered in mice by HBcAg176~185 during exposure to HBV is important in the determination of HBV persistent infection.

Although it has been reported that PD-1 is associated with apoptosis [18,19], inhibition of TNF-α led to higher maintained serum HBV viral load with an increased number of intrahepatic PD-1(high)CD127(low)-exhausted T-cells, which resulted in the reduction of HBV clearance in the mouse model [20]. Treatment with monoclonal antibodies of PD-1 might be useful for patients with HBV reactivation, especially those treated with immune-suppressing and anti-cancer drugs, as reactivation is now becoming a global issue [21,22,23,24,25].

NVR 3–778, a candidate HBV core inhibitor, disrupts the HBV lifecycle by inducing the assembly of defective viral capsids, but it is also a potent inhibitor of HBV replication in both cell culture models and a humanized albumin enhancer/promoter urokinase plasminogen activator (UPA)/severe combined immunodeficiency (SCID) mouse model of HBV infection [26,27]. Treatment with NVR 3–778 for six weeks resulted in the suppression of HBV DNA replication. The efficacy of NVR 3–778 was superior to peginterferon and was similar to the nucleoside analog, entecavir, in a humanized UPA/SCID mouse model [27]. The phase 1a clinical trial of NVR 3–778 was completed in New Zealand in 2014, and the phase 1b clinical trial of NVR 3–778 is currently enrolling patients [26].

Previously, a single CTL epitope (HBV core 18–27: FLPSDFFPSV) vaccine CY-1899 was used to treat patients with chronic hepatitis B [28]. Although CY-1899 initiated CTL activity, low-level CTL activity does not seem to be associated with HBV clearance [28]. Epitopes of both HBsAg and HBV core were loaded on dendritic cells, and epitope-pulsed dendritic cells were used in chronic hepatitis B patients. The results of the epitope study demonstrated the potent antiviral effects of dendritic cell vaccines that contain HBsAg and HBV core epitopes [29]. A phase III clinical trial of an HBsAg/HBV core-based therapeutic vaccine, administered through the nasal and subcutaneous routes, is currently underway in chronic hepatitis B patients [29].

## 5. Conclusions

Hepatic inflammation induced by interferons and cytokines is associated with hepatocarcinogenesis [30]. The HBV core protein could produce a response through interferon and inflammatory cytokines to induce hepatic inflammation and, possibly, hepatocarcinogenesis, although HBe antigen (HBeAg) inhibits cytokine production and prevents persistent HBV infection [31,32]. Thus, inhibitors of HBV core may be useful for preventing the progression of liver diseases in patients infected with HBV.
